# *Osx-Cre* Targets Multiple Cell Types besides Osteoblast Lineage in Postnatal Mice

**DOI:** 10.1371/journal.pone.0085161

**Published:** 2014-01-15

**Authors:** Jianquan Chen, Yu Shi, Jenna Regan, Kannan Karuppaiah, David M. Ornitz, Fanxin Long

**Affiliations:** 1 Department of Orthopaedic Surgery, Washington University School of Medicine, St. Louis, Missouri, United States of America; 2 Department of Developmental Biology, Washington University School of Medicine, St. Louis, Missouri, United States of America; 3 Department of Medicine, Washington University School of Medicine, St. Louis, Missouri, United States of America; University of Frankfurt - University Hospital Frankfurt, Germany

## Abstract

Osterix (Osx or Sp7) is a zinc-finger-family transcriptional factor essential for osteoblast differentiation in mammals. The *Osx-Cre* mouse line (also known as Osx1-GFP::Cre) expresses GFP::Cre fusion protein from a BAC transgene containing the *Osx* regulatory sequence. The mouse strain was initially characterized during embryogenesis, and found to target mainly osteoblast-lineage cells. Because the strain has been increasingly used in postnatal studies, it is important to evaluate its targeting specificity in mice after birth. By crossing the *Osx-Cre* mouse with the *R26-mT/mG* reporter line and analyzing the progenies at two months of age, we find that *Osx-Cre* targets not only osteoblasts, osteocytes and hypertrophic chondrocytes as expected, but also stromal cells, adipocytes and perivascular cells in the bone marrow. The targeting of adipocytes and perivascular cells appears to be specific to those residing within the bone marrow, as the same cell types elsewhere are not targeted. Beyond the skeleton, Osx-Cre also targets the olfactory glomerular cells, and a subset of the gastric and intestinal epithelium. Thus, potential contributions from the non-osteoblast-lineage cells should be considered when *Osx-Cre* is used to study gene functions in postnatal mice.

## Introduction

Osterix (Osx or Sp7) is a zinc finger family transcriptional factor critical for osteoblast differentiation[1]. During embryonic skeletal development, Osx is initially expressed in the perichondrium flanking the hypertrophic cartilage, where osteoblasts first arise to produce the bone collar (cortical bone). Later during development, the perichondrial Osx-expressing osteoprogenitors co-migrate with the blood vessels that invade the hypertrophic cartilage, and to generate osteoblasts responsible for depositing the trabecular bone [2], [3]. In addition, Osx is also detected in early hypertrophic chondrocytes at a relatively weak level [4]. Genetic studies have revealed the essential role of Osx in osteoblast differentiation[5]. In Osx-null embryos, cartilage elements are largely normal but osteoblast differentiation fails to complete, resulting in a complete lack of bone tissue [1]. In these embryos, *Runx2* expression is relatively normal, but other osteoblast markers including *Col1a1*, *Bsp*, and *osteocalcin* are either absent or severely suppressed [1]. On the other hand, the expression of *Osx* is abolished in *Runx2*-null embryos[1]. Thus, Osx functions genetically downstream of Runx2 to control osteoblast differentiation[6]. In addition to its role in embryonic osteoblast differentiation, Osx also plays a critical role in the formation and function of postnatal osteoblast and osteocyte [7].

The Cre/loxP technology enables gene deletion in specific cell types and has significantly advanced our understanding of gene functions in both physiological and pathological conditions. In this system, specificity is achieved by expression of the Cre recombinase under the control of cell type-specific regulatory sequences. *Osx-Cre* (*Osx1*-*GFP::Cre*), a BAC transgenic mouse line expressing a GFP::Cre fusion protein from the regulatory sequence of *Osx*, was generated to direct gene deletion in the osteoblast lineage [8]. The initial characterization of this mouse line revealed that Cre activity is largely restricted to the osteogenic perichondrium, periosteum and osteoblast-lineage cells within the marrow cavity, but that analysis was limited to the embryo [8]. Although in recent years the *Osx-Cre* mouse line has been increasingly used to study the osteoblast lineage in postnatal mice [9], [10], [11], [12], [13], the targeting specificity of *Osx-Cre* in postnatal bones is yet to be formally evaluated.

Here we assess the cell types targeted by Osx-Cre in two-month-old mice by monitoring GFP expression from the *R26-mT/mG* reporter allele. The *R26-mT/mG* allele ubiquitously expresses a membrane-targeted red fluorescent protein (mTomato) but switches to expressing a membrane-targeted green fluorescent protein (mGFP) upon Cre recombination. We find that within the skeleton, *Osx-Cre* targets not only osteoblast lineage cells and a subset of chondrocytes, but also stromal cells, adipocytes and perivascular cells specifically within the bone marrow. Moreover, *Osx-Cre* also targets cells within the olfactory bulb, the intestine and the stomach.

## Materials and Methods

### Mouse strains

*Osx-Cre (Osx1*-*GFP::Cre)* and *R26-mT/mG* mouse lines are as previously described [8], [14]. All mouse procedures used in this study were approved by the Animal Studies Committee at Washington University.

### Cryostat sections

Two-month-old mice were perfused with 4% PFA as described previously[15]. After perfusion, tibias were dissected and fixed in 4% PFA at 4°C overnight. The fixed tibias were decalcified in 14% EDTA for 3 days and then snap-frozen in OCT embedding medium. Frozen sections were cut at 8 µm thickness with a cryostat equipped with Cryojane (Leica, IL). The sections were kept at −20°C until analyses.

### Immunofluorescence staining

For detection of GFP, perilipin or αSMA, immunostaining was performed on cryostat sections using a chicken polyclonal GFP antibody (1∶2500; Abcam, Cambridge, MA), or rabbit monoclonal perilipin antibody (1∶100; Cell Signaling Technology, Danvers, MA), or mouse monoclonal αSMA antibody (1∶500, Sigma, St. Louis, MO). The secondary antibodies are as follows: Alexa Fluor® 488 Goat Anti-Chicken IgG (for GFP); Alexa Fluor® 647 F(ab')_2_ Fragment of Goat Anti-Rabbit IgG (for perilipin), and Alexa Fluor® 647 Goat Anti-Mouse IgG_2a_ (for αSMA) (all at 1∶250, Life Technologies, Grand Island, NY). Sections were mounted with VECTASHIELD Mounting Medium containing DAPI (VECTOR LABORATORIES, Burlingame, CA).

### Quantitative analyses

All quantitative data were obtained from three independent animals. Statistical analyses were performed with student's t-test.

## Results

### *Osx-Cre* targets osteoblasts, osteocytes, bone marrow stromal cells and hypertrophic chondrocytes

**Figure 1:**
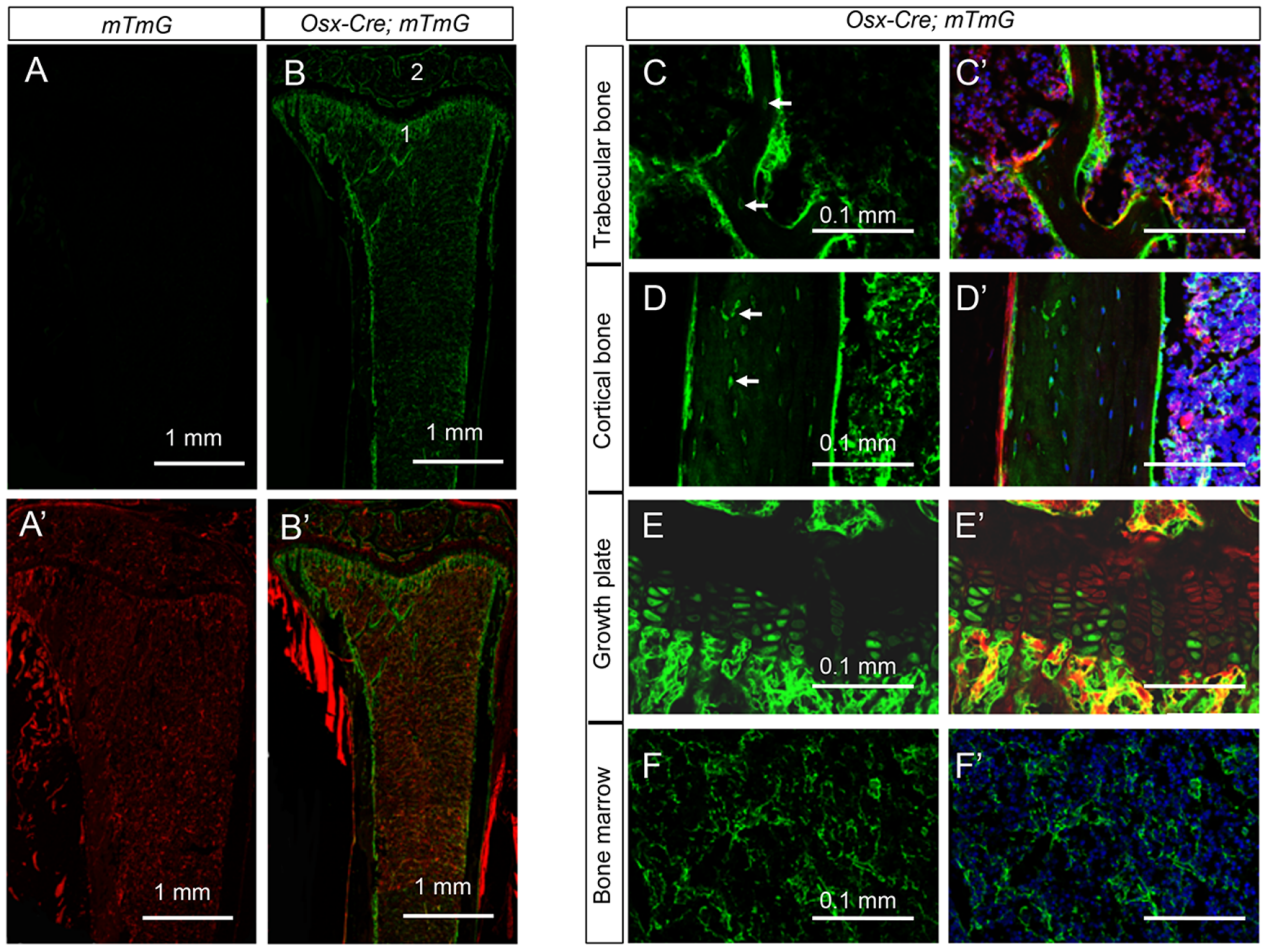
*Osx-Cre* targets osteoblast lineage cells, hypertrophic chondrocytes and bone marrow stromal cells. (A–B) Confocal images for direct fluorescence from EGFP (A, B) or EGFP/tdTomato (A′, B′) on longitudinal tibial sections from two-month-old *R26-mT/mG* (A, A′) or *Osx-Cre; R26-mT/mG* mice (B, B′); 1: chondro-osseous junction of primary ossification center; 2: secondary ossification center. (C–F, C′–F′) Higher magnification images for EGFP (C–F) or EGFP/tdTomato: trabecular bone (C, C′), cortical bone (D, D′), growth plate (E, E′) and bone marrow (F, F′). Red: membrane-targeted tdTomato; Green: membrane-targeted EGFP; Blue: DAPI. Arrow: osteocyte.

To characterize the targeting specificity of *Osx-Cre* in postnatal mouse bones, we generated *Osx-Cre; R26-mT/mG* mice (one copy each of Osx-Cre and R26-mT/mG) and analyzed GFP expression on sections of long bones at two months of age. As expected, bone sections from the control *R26-mT/mG* mice did not exhibit any GFP (Fig. 1A, A′), but those from *Osx-Cre; R26-mT/mG* mice contained many GFP-positive cells (Fig. 1B, B′). Consistent with the targeting of osteoblast-lineage cells, many GFP-positive cells were present at both primary and secondary ossification centers, as well as the cortical bone surfaces (Fig. 1B, B′). Examination at a higher magnification revealed that essentially all cells associated with the trabecular, endosteal and periosteal surfaces, as well as most osteocytes were GFP-positive (Fig. 1C, C′, D, D′). Moreover, a large population of bone marrow stromal cells expressed GFP (Fig. 1F, F′). These cells exhibited a reticular morphology and were readily distinguishable from the hematopoietic population. Finally, GFP was detected in some prehypertrophic and hypertrophic chondrocytes within the growth plate (Fig. 1E, E′), consistent with previous reports of endogenous *Osx* expression in these cells [16]. Thus, in addition to osteoblasts and osteocytes, *Osx-Cre* also targets the bone marrow stromal cells and the growth plate hypertrophic chondrocytes in postnatal mice.

### *Osx-Cre* targets adipocytes specifically in the bone marrow

**Figure 2:**
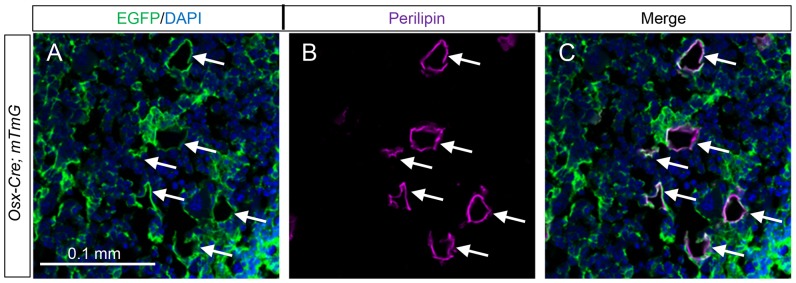
*Osx-Cre* marks adipocytes in bone marrow. (A–B) Double immunostaining for EGFP (A) and perilipin (B) on longitudinal sections of tibias from two-month-old *Osx-Cre; R26-mT/mG* mice. (C) Co-localization of EGFP and perilipin. Arrows denote co-expression of GFP and perilipin. Green: EGFP; magenta: perilipin; blue: DAPI.

**Figure 3:**
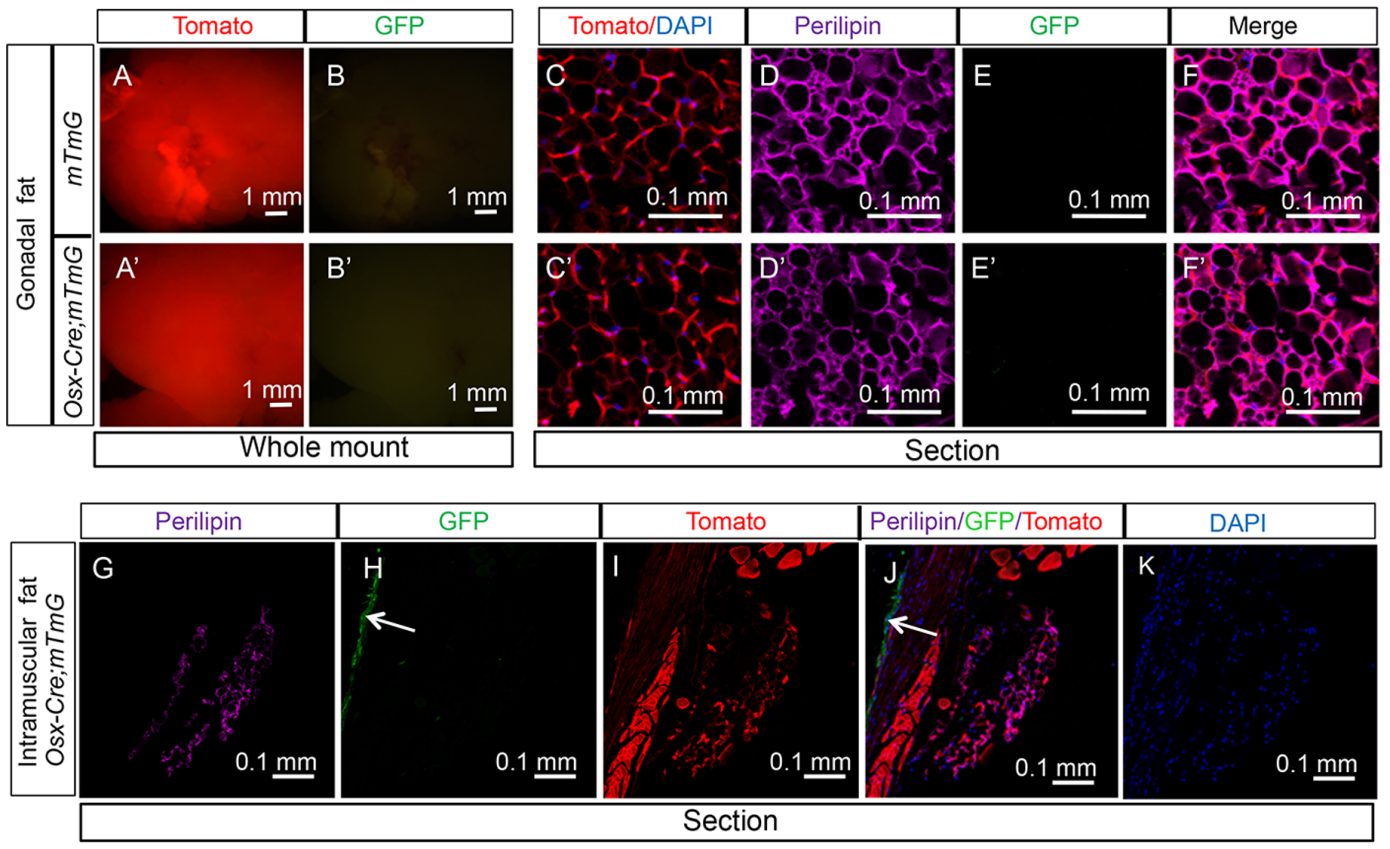
*Osx-Cre* does not mark non-bone marrow adipocytes. (A–B) Images for direct fluorescence from tdTomato (A) or EGFP (B) in whole-mount gonadal fat depots from two-month-old *Osx-Cre*; *R26-mT/mG* mice. (C–F) Direct fluorescence for tdTomato (C) and immunofluorescence for perilipin (D) and EGFP (E) on sections of gonadal fat depots from two-month-old *Osx-Cre;R26-mT/mG* mice. (G–K) Imaging of longitudinal sections of an intramuscular fat depot associated with a tibia from two-month-old *Osx-Cre;R26-mT/mG* mice. G: perilipin immunofluorescence; H: EGFP immunofluorescence; I: direct fluorescence for tdTomato; J: merged view of G–I; K: DAPI staining. Arrow: GFP-positive periosteum.

Within the bone marrow, in addition to the reticular stromal cells, we also detected other GFP-positive cells that appeared to be adipocytes. To confirm this observation, we performed double immunofluorescence experiments with antibodies against GFP and the adipocyte-specific marker perilipin. Indeed, on bone sections from the *Osx-Cre; R26-mT/mG* mice, more than 90% of the perilipin-positive adipocytes present within the bone marrow also stained for GFP (Fig. 2). To determine whether *Osx*-*Cre* also targets adipocytes outside the bone marrow, we examined the whole-mount gonadal fat depot from *Osx-Cre; R26-mT/mG* mice under a fluorescence microscope but didn't detect any GFP (Fig. 3A–B). Double immunostaining of sections from the gonadal fat depot confirmed that the perilipin-positive adipocytes were GFP-negative (Fig. 3C–F). Similarly, adipose tissues adjacent to the periosteum and associated with the skeletal muscle did not express GFP (Fig.3G–J). Thus, *Osx-Cre* effectively targets adipocytes specifically within the bone marrow environment.

### *Osx-Cre* targets perivascular cells specifically in the bone marrow

**Figure 4:**
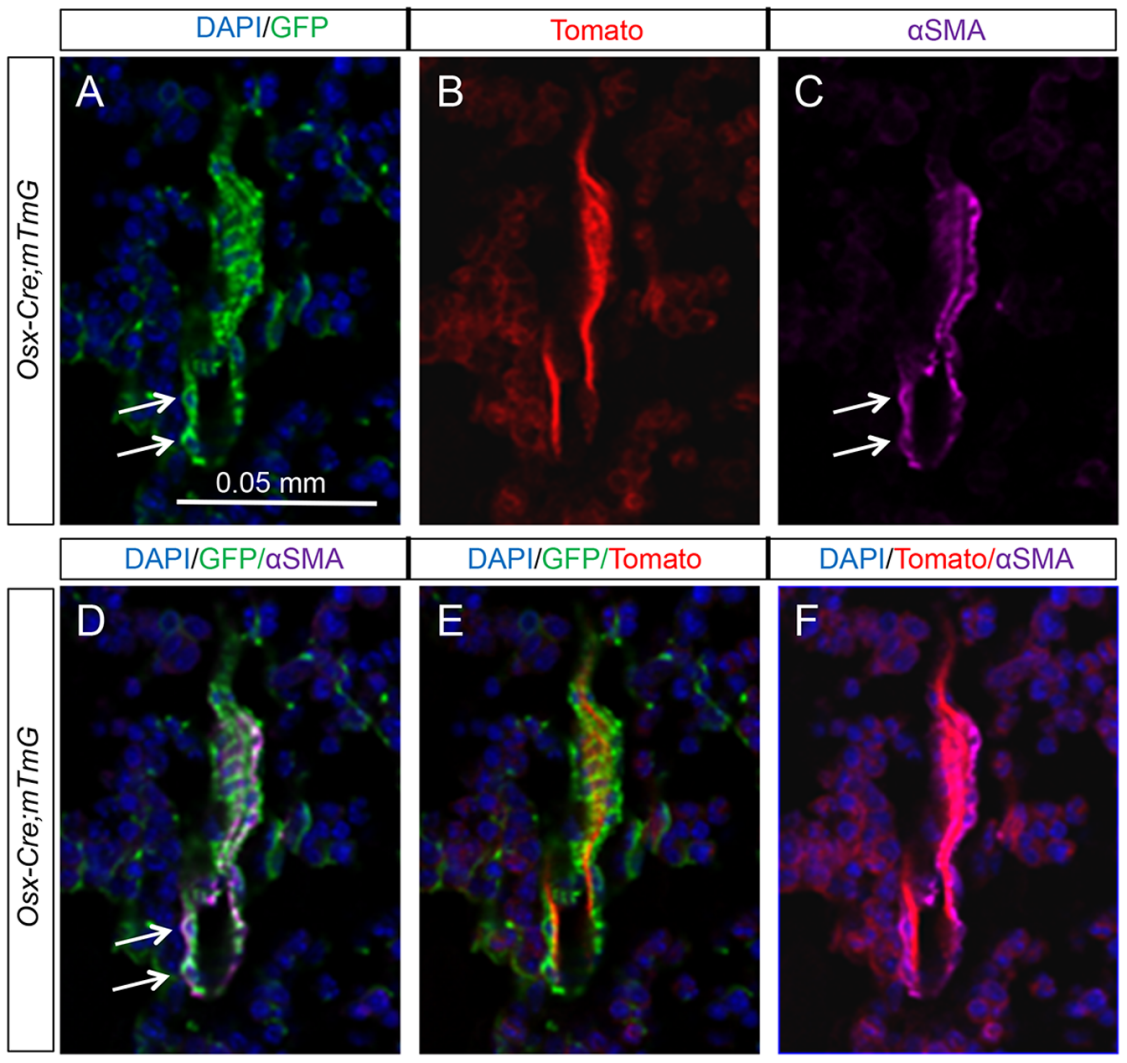
Osx-Cre marks perivascular smooth muscle cells in bone marrow. (A–C) Confocal images of EGFP (A), tdTomato (B), and αSMA (C) on longitudinal sections of tibias from two-month-old *Osx-Cre; R26-mT/mG* mice. (D–F) Merged images. Arrow: co-expression of EGFP and αSMA. EGFP and αSMA: immunofluorescence; tdTomato: direct fluorescence. DAPI stains DNA blue.

Our initial examination of the long bone sections revealed that certain GFP-positive cells appeared to associate directly with blood vessels. To confirm the identity of these cells as perivascular smooth muscle cells, we performed double immunofluorescence experiments with antibodies against GFP and αSMA. Direct visualization of tdTomato under a fluorescence microscope revealed that the endothelial cells expressed a strong signal, whereas the αSMA-positive cells were immediately adjacent to the endothelial cells as expected (Fig. 4F). Importantly, in three *Osx-Cre; R26-mT/mG* mice analyzed, nearly all αSMA-positive perivascular smooth muscle cells co-expressed GFP (Fig. 4D) (93.9±2.8%, n = 3), and 100% of the ten bone marrow blood vessels observed were found to associate with GFP-positive cells. In contrast, the blood vessels abundantly present in the gonadal adipose tissue did not contain GFP-positive cells (Fig. 3C′, E′). Thus, *Osx-Cre* targets a high percentage of the perivascular smooth muscle cells specifically within the bone marrow.

### *Osx-Cre* targets cells beyond the skeleton

**Figure 5:**
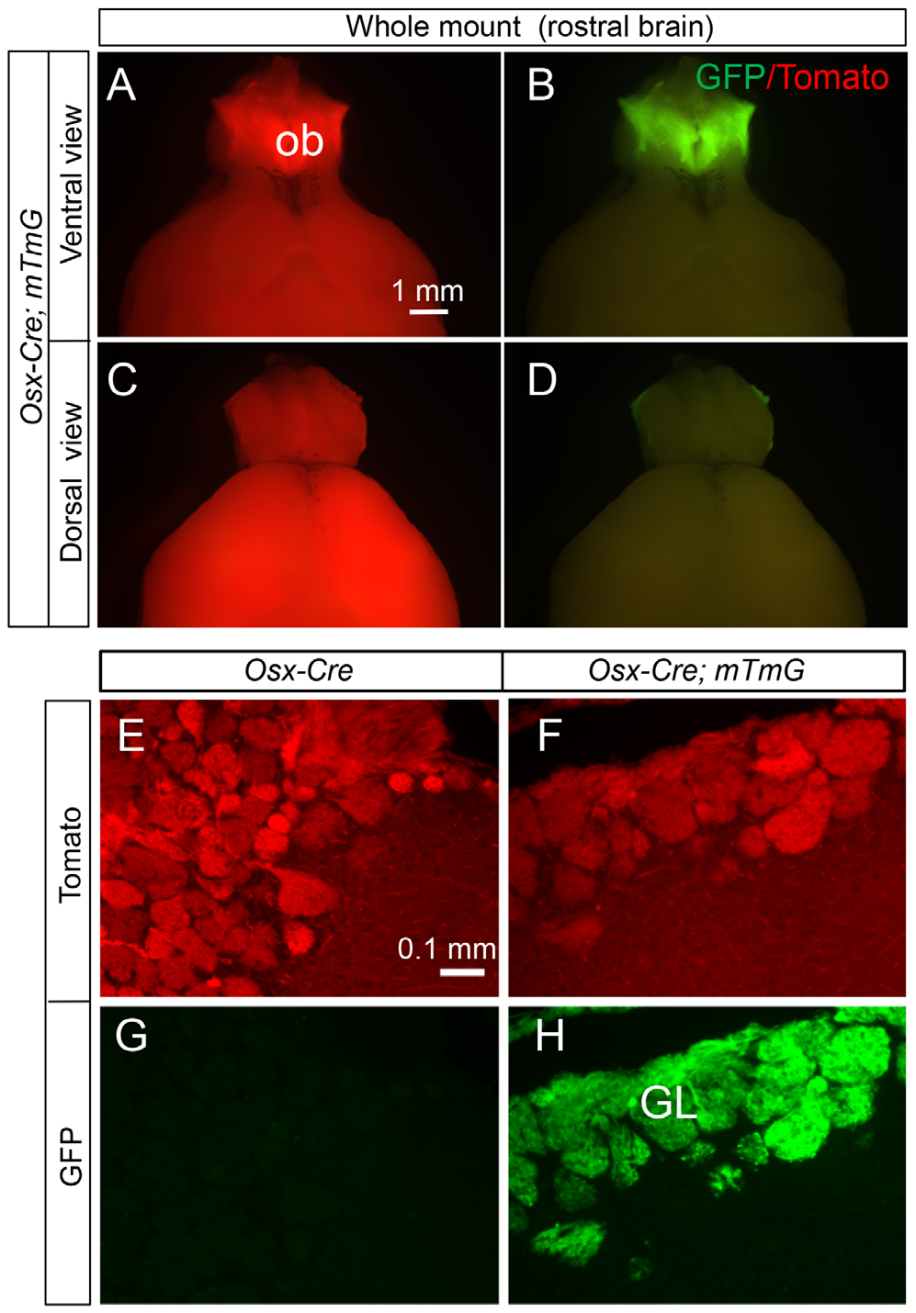
Osx-Cre targets olfactory glomerular cells. (A–D) Direct fluorescence of whole-mount rostral brain from a two-month-old *Osx-Cre; R26-mT/mG* mouse. ob: olfactory bulb. (E–H) Direct fluorescence of tdTomato (E, F) and EGFP (G, H) on sagittal sections through the olfactory bulb of *Osx-Cre* (E, G) or *Osx-Cre; R26-mT/mG* (F, H) mice at two months of age. GL: glomerular layer.

**Figure 6:**
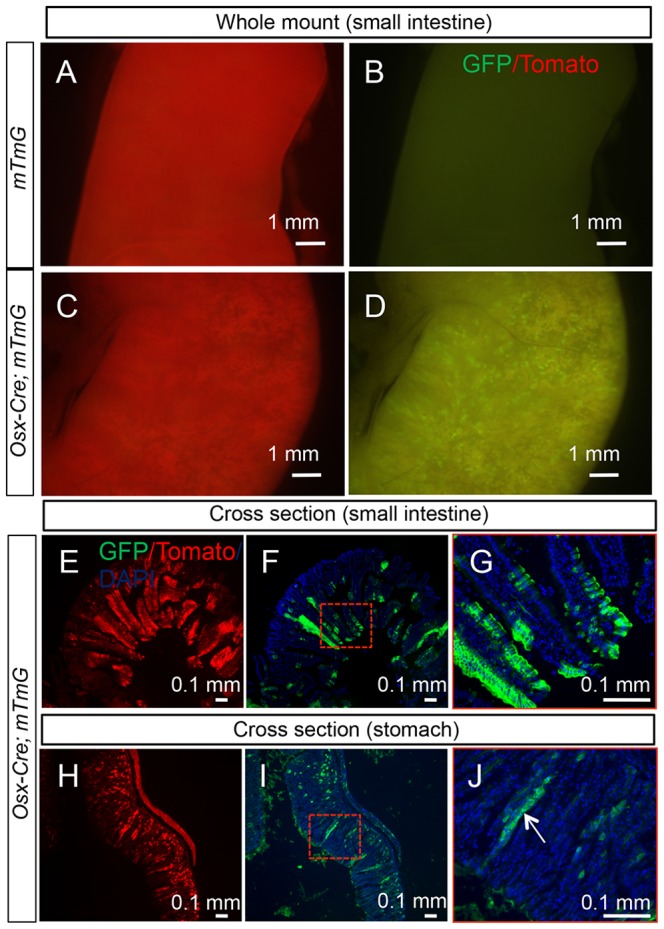
Osx-Cre targets gastric and intestinal epithelia. (A–D) Direct fluorescence of tdTomato (A, C) and EGFP (B, D) in whole-mount small intestine of two-month-old R26-mT/mG (A, B) or Osx-Cre; R26-mT/mG mice (C, D). (E–G) Direct fluorescence on cross sections of small intestine from a two-month-old Osx-Cre; R26-mT/mG mouse. Red-boxed area in F shown at a higher magnification in G. (H–J) Direct fluorescence on stomach sections from a two-month-old Osx-Cre; R26-mT/mG mouse. Red-boxed area in I shown at a higher magnification in J. White arrow denotes parietal cells.

We next examined all tissues of the *Osx-Cre; R26-mT/mG* mouse for potential targeting by *Osx-Cre*. We first ensured that no GFP was detected in any of the organs of the control *R26-mT/mG* mice. In *Osx-Cre; R26-mT/mG* mice, we found no GFP in liver, kidney, pancreas, heart, lung, adrenal gland, thymus, thyroid or skeletal muscle. Much of the brain was also negative, but the olfactory bulb expressed strong GFP as evident from the ventral view of the rostral brain (Fig. 5A–D). Sagittal sections through the bulb revealed that *Osx-Cre* targeted essentially all cells of the glomerular layer (Fig. 5E, F). In addition, a strong GFP signal was observed in whole-mount samples of the stomach, small and large intestines from *Osx-Cre; R26-mT/mG* but not *R26-mT/mG* littermates (Fig. 6 A–D, data not shown). Cross-section of the small intestine revealed that GFP was non-uniformly expressed by enterocytes along the villi, whereas cells in the crypts were largely negative (Fig. 6E–G). Similarly, cross sections through the stomach indicated mosaic GFP expression in the epithelium including parietal cells (Fig. 6 H–J). The precise identity of the targeted intestinal or gastric cells was not further pursued in the present study.

## Discussion

### *Osx-Cre* and bone marrow mesenchymal stem cells

Evidence suggests that mesenchymal stem cells (MSCs) residing within the adult bone marrow produce osteoblasts, adipocytes and bone marrow stromal cells necessary for postnatal tissue homeostasis [17], [18]. Moreover, accumulating evidence supports that mesenchymal stem cells reside within a perivascular niche [18], [19], [20], [21], [22]. Our present work demonstrates that *Osx-Cre* targets not only osteoblasts and osteocytes, but also the bone marrow stromal cells and perivascular smooth muscle cells, even though the later cell types do not actively express the GFP::Cre protein. These results raise the possibility that *Osx-Cre* targets a common progenitor of the aforementioned cell types, perhaps the bone marrow MSC. Alternatively, *Osx-Cre* may mark a diverse group of progenitors each producing a single mature cell type (e.g., stromal or perivascular cell). In either case, the progenitors do not appear to continuously express *Osx* or *Osx-Cre* throughout postnatal life, as cells actively expressing *Osx* postnatally, when marked with *Osx-CreER* in response to tamoxifen, failed to sustain the turnover of either osteoblasts or stromal cells [15], [23]. Overall, the current data support the model that embryonic *Osx*-expressing cells give rise to the bone marrow mesenchymal progenitors in postnatal mice. Additional experiments are necessary to test this hypothesis formally.

### Organ-specific origin of bone marrow adipocytes and perivascular cells

Bone marrow adipose tissue is believed to be metabolically different from non-marrow peripheral fat depots [24]. Our data showed that bone marrow but not other adipocytes are derived from Osx-lineage cells, indicating a distinct cell origin of the bone marrow adipocytes. Similarly, perivascular smooth muscle cells specifically associated with bone marrow but not other blood vessels are derived from *Osx-*expressing progenitors. While this work was in review, others using the Ai9 Cre reporter mouse reported similar findings about the targeting of bone marrow adipocytes and perivascular smooth muscle cells by *Osx-Cre*, thus allaying the concern over limitations of a single Cre reporter [25]. Overall, these findings support the view that mesenchymal cell types in different organs may be derived from organ-specific stem/progenitor cells that reside locally.

### Limitation of *Osx-Cre* as a tool for studying osteoblast biology

Our data clearly indicate that *Osx-Cre*, when activated in the embryo, targets more than osteoblast-lineage cells in postnatal mice. These findings echo the increasing concern that many Cre strains exhibit some degree of unintended recombination activity [26]. Whereas the relationship between marrow fat and bone is increasingly appreciated [24], the potential influence of stromal cells and perivascular cells on bone is largely unknown. In addition, *Osx-Cre* targets olfactory glomerular cells and the GI tract epithelia. Targeting of the olfactory bulb is consistent with endogenous *Osx* expression as previously reported in this organ [27]. On the other hand, it is not clear at present whether *Osx* is normally expressed in the GI tract, or the mosaic Cre activity there simply reflects a phenomenon specific to the *Osx-Cre* transgene. Furthermore, it is not known whether *Osx-Cre*-mediated gene deletion in the olfactory bulb or the GI track affects bone physiology. Nonetheless, caution needs to be taken when one interprets postnatal bone phenotypes caused by gene deletion with *Osx-Cre* beginning in the embryo.
